# Identification of FKBP10 prognostic value in lung adenocarcinoma patients with surgical resection of brain metastases: A retrospective single-institution cohort study

**DOI:** 10.1016/j.clinsp.2023.100212

**Published:** 2023-05-16

**Authors:** Zhi-Dan Liu, Song-Quan Wang, Sai Li, Jie He, Shao-Hua Wang, Hong-Qing Cai, Jing-Hai Wan

**Affiliations:** a Department of Neurosurgery, The Second Affiliated Hospital, Anhui Medical University, Hefei, China; b Department of Neurosurgery, National Cancer Center/National Clinical Research Center for Cancer/Cancer Hospital, Chinese Academy of Medical Sciences and Peking Union Medical College, Beijing, China; c Department of Neurosurgery, Second Hospital of Shanxi Medical University Taiyuan, China; d Department of Neurosurgery, Fuyang Hospital of Anhui Medical University, Fuyang, China

**Keywords:** Brain metastases, Lung adenocarcinoma, FKBP10

## Abstract

**Objective:**

To explore the expression levels and clinical value of FKBP10 in lung adenocarcinoma brain metastases.

**Design:**

A retrospective single-institution cohort study.

**Patients:**

The perioperative records of 71 patients with lung adenocarcinoma brain metastases who underwent surgical resection at the authors’ institution between November 2012 and June 2019 were retrospectively analyzed.

**Methods:**

The authors evaluated FKBP10 expression levels using immunohistochemistry in tissue arrays of these patients. Kaplan-Meier survival curves were constructed, and a Cox proportional hazards regression model was used to identify independent prognostic biomarkers. A public database was used to detect FKBP10 expression and its clinical value in primary lung adenocarcinoma.

**Results:**

The authors found that the FKBP10 protein was selectively expressed in lung adenocarcinoma brain metastases. Survival analysis showed that FKBP10 expression (p = 0.02, HR = 2.472, 95% CI [1.156, 5.289]), target therapy (p < 0.01, HR = 0.186, 95% CI [0.073, 0.477]), and radiotherapy (p = 0.006, HR = 0.330, 95% CI [0.149, 0.731]) were independent prognostic factors for survival in lung adenocarcinoma patients with brain metastases. The authors also detected FKBP10 expression in primary lung adenocarcinoma using a public database, found that FKBP10 is also selectively expressed in primary lung adenocarcinoma, and affects the overall survival and disease-free survival of patients.

**Limitations:**

The number of enrolled patients was relatively small and patients’ treatment options varied.

**Conclusions:**

A combination of surgical resection, adjuvant radiotherapy, and precise target therapy may benefit the survival of selected patients with lung adenocarcinoma brain metastases. FKBP10 is a novel biomarker for lung adenocarcinoma brain metastases, which is closely associated with survival time and may serve as a potential therapeutic target.

## Highlights

• FKBP10 proteins are highly expressed in lung adenocarcinoma, including brain metastasis tissue.
• FKBP10 is a valuable prognostic indicator for lung adenocarcinoma.
• FKBP10 is a potential clinical biomarker for lung adenocarcinoma.

## Introduction

Lung cancer is the second most commonly diagnosed cancer worldwide.^1^ As the overall survival of lung cancer continues to improve following initial cancer diagnoses, the incidence of brain metastases increases.^2^^,^^3^ Moreover, lung cancer is reported as the most common origin of brain metastases, and lung adenocarcinoma is the largest subgroup.^2^^,^^4^^,^^5^ It has been reported that about 25%–50% of lung cancer patients will develop brain metastases during treatment.^6^ Surgical resection, stereotactic radiosurgery, and whole-brain radiation therapy are the primary treatment modalities.^7^ With the development of new technology, targeted agents and immune checkpoint inhibitors have also been used in lung cancer patients with brain metastases. However, the prognosis of these patients is still poor, and brain metastases limit the survival time of patients with lung cancer.^2^^,^^8^ Treatment of lung adenocarcinoma brain metastases is a challenge for clinicians. Increased molecular understanding of brain metastases will encourage the continued development of novel targeted therapies that have higher bioavailability beyond the blood–tumor barrier. Thus, there is an urgent need to identify the molecular alterations involved in lung adenocarcinoma cells and explore possible targets for the clinical treatment of this disease.

FK506-Binding Protein 10 (FKBP10), a member of the FKBP family encodes a 65 kDa protein located in the rough endoplasmic reticulum and is involved in collagen biosynthesis through collagen pyridinoline cross-linking and construction of bone and tendons.^9^^,^^10^ The authors have previously reported that FKBP10 is overexpressed in glioma and is involved in the proliferation of glioma cells by interacting with Hsp47 and activating AKT-CREB-PCNA signaling pathways.^11^ In addition, FKBP10 has also been detected in human colorectal adenocarcinoma and gastric cancer but not in healthy colorectal and gastric tissues.^12^^,^^13^ It has also been reported that FKBP10 is a cancer-selective molecule with a key role in translational reprogramming, stem-like traits, and growth of lung cancer.^14^ A previous study demonstrated that FDA-approved nintedanib for Idiopathic Pulmonary Fibrosis (IPF) therapy could downregulate FKBP10 expression in IPF fibroblasts.^15^ Thus, FKBP10 plays an important role in cancer development and may serve as a potential target for therapeutic drugs. However, the protein expression and clinical value of FKBP10 in brain metastases of lung adenocarcinoma remain unclear.

In the present study, the authors detected the role of FKBP10 in lung adenocarcinoma brain metastases, analyzed survival, and found that FKBP10 was highly expressed in lung adenocarcinoma brain metastases, which has an independent prognostic impact on these patients.

## Methods and materials

### Patients and tissue samples

A single-institution retrospective review included lung adenocarcinoma patients with brain metastases in the department as follows. (1) Patients with solitary or oligometastatic lung adenocarcinoma brain metastasis lesions, which can be surgically removed via a single approach. (2) Patients having a Karnofsky score ≥ 70. (3) Patients were fit to undergo surgery as assessed by the anesthesia team. (4) Primary and extracranial lesions were stable. (5) Informed consent was provided.

71 patients with surgical resection for lung adenocarcinoma brain metastases were retrospectively enrolled in the Department of Neurosurgery at the hospital between November 2012 and June 2019. Tumor tissues were obtained from the Department of Pathology and anonymized with patient information and diagnosis during the experimental phase. Fourteen samples had matched adjacent non-neoplastic tissues collected from the edema-affected tissues surrounding the metastases or resected in the process of obtaining deep-seated metastases. All patients were routinely reviewed with brain enhanced MRI every three months after neurosurgery. The follow-up information was confirmed by telephone and outpatient before September 2022. The clinical and radiographic information was acquired for all 71 patients with lung adenocarcinoma brain metastases. Written informed consent was obtained from all patients for sampling and research. This study was approved by the ethics committee of the authors’ institution.

### Data collection from public databases

KMPLOT^16^ and CANCERTOOL^17^ were used to investigate the relevance of FKBP10 in human primary lung adenocarcinoma. These two public databases catalog the correlation between patient survival and the expression of individual genes in several types of cancer, including lung cancer.

### Tissue microarray, immunohistochemistry, and evaluation of immunostaining

A tissue microarray was constructed as previously described.^18^ The constructed tissue microarrays were cut into 5-μm sections. Immunohistochemistry was performed as described previously^18^ using anti-FKBP10 antibody (1:2000, 50353, Sigma). A NanoZoomer (Hamamatsu, Japan) high-resolution scanner was used to scan the immunostaining slides. Two pathologists evaluated the immunostaining blinded to clinical information. Immunostaining of FKBP10 was assessed according to staining intensity and the percentage of immunoreactive cells. The intensity of immunostaining was graded as 0 (no staining), 1 (weak staining), 2 (moderate staining), and 3 (strong staining). The percentage of immunoreactive cells was graded as 0 (0%), 1 (1%–25%), 2 (26%–50%), 3 (51%–75%), or 4 (76%–100%). The total score for FKBP10 immunostaining was calculated using the following formula: intensity of immunostaining ×  percentage of immunoreactive cells. The authors defined high FKBP10 expression as a total score ≥ 4 and low FKBP10 expression as a total score < 4. A score of zero represents a negative expression of FKBP10.

### Statistical analysis

All data were analyzed using SPSS Statistics software (version 22.0) and GraphPad Prism 5.0. The χ^2^ test was used to assess the relationship between molecular alterations and clinicopathological parameters. Kaplan-Meier survival curves were constructed, and differences were detected using the log-rank test or the Breslow-Wilcoxon test. A Cox proportional hazards regression model was used to identify independent prognostic biomarkers for lung adenocarcinoma brain metastases. Differences with a 2-sided p<0.05 were considered statistically significant.

## Results

### FKBP10 proteins is selectively expressed in lung adenocarcinoma brain metastases

**Figure 1:**
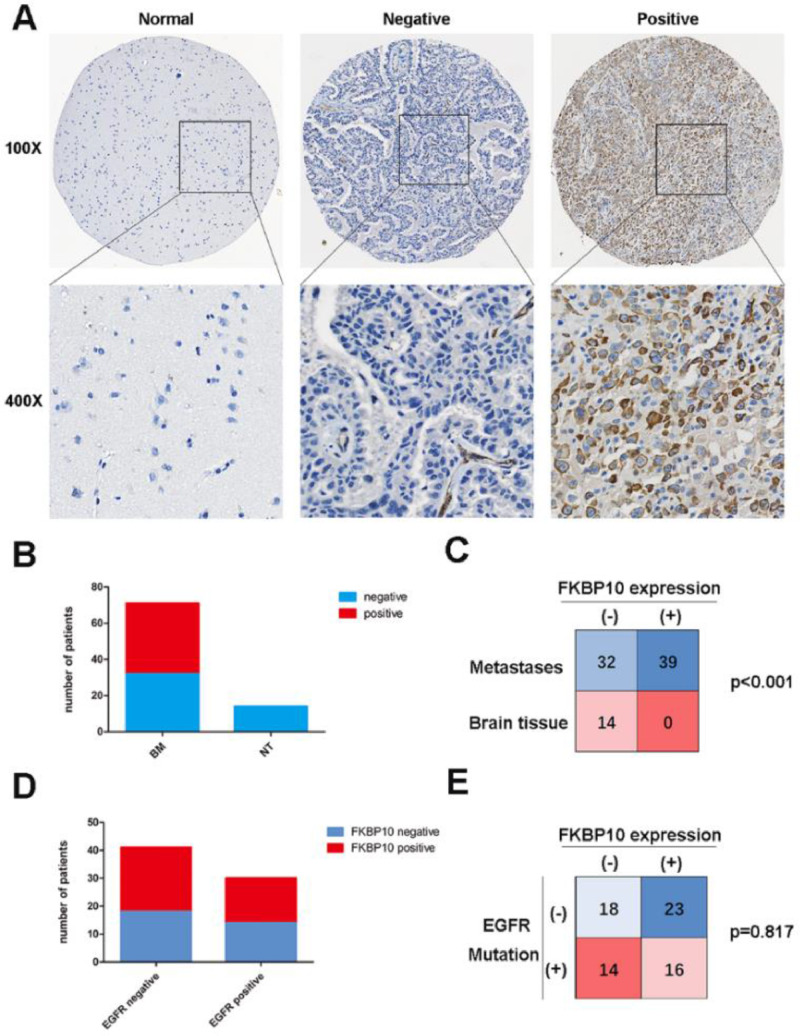
FKBP10 expression in lung adenocarcinoma brain metastases and non-neoplastic brain tissues. (A) Representative images of FKBP10 immunostaining in non-neoplastic tissues and lung adenocarcinoma brain metastases tissues. (B) FKBP10 expression in brain metastases and non-tumor tissues. (C) Number of cases and percentage of FKBP10 positive and negative expression in lung adenocarcinoma brain metastases and non-neoplastic brain tissues. (D) In our cohort, expression of FKBP10 in brain metastases with EGFR mutations vs non-mutants, respectively. (E) Number of cases and percentage of FKBP10 positive and negative expression in lung adenocarcinoma brain metastases with EGFR mutations and non-mutants.

To determine whether FKBP10 is exclusively expressed in lung adenocarcinoma brain metastases tissue, the authors performed FKBP10 Immunohistochemistry (IHC) analysis in 14 cases of non-tumor brain tissue and 71 cases of lung adenocarcinoma brain metastases. FKBP10-positive cells were detected in 39 patients (55%) with lung adenocarcinoma brain metastases. No FKBP10-positive cells were found in the non-tumor brain tissue (Fig. 1 A, B and C). The difference in FKBP10 positive rate between lung adenocarcinoma brain metastases tissue and non-tumor brain tissue was significant (p < 0.001), which indicated that FKBP10 expression is tumor-specific in the cohort of patients. In addition, the authors compared FKBP10 expression between different subgroups divided by sex, age, targeted therapy, and radiotherapy. The outcomes are presented in Table 1. The authors found that elder patients (≥ 60 years) were more likely to have high FKBP10 expression (p = 0.006).

**Table 1:** Relationship between FKBP10 expression and clinicopathologic parameters in 71 lung adenocarcinoma brain metastases patients.

Variable	N°	FKBP10 low expression	FKBP10 high expression	p-value
Gender				
Male	41	20 (48.8%)	21 (51.2%)	
Female	30	19 (63.3%)	11 (36.7%)	0.233
Age (years)				
≥60	36	14 (38.9%)	22 (61.1%)	
<60	35	25 (71.4%)	10 (28.6%)	0.006^a^
Adjuvant radiotherapy				
No	48	27 (56.3%)	21 (43.7%)	
Yes	23	12 (52.2%)	11 (47.8%)	0.747
Target therapy				
No	41	20 (48.8%)	21 (51.2%)	
Yes	30	19 (63.3%)	11 (36.7%)	0.223

a p < 0.05 was considered statistically significant.

### The relationship between FKBP10 expression and other biomarkers

Epidermal Growth Factor Receptor (EGFR) mutation is a common biomarker and therapy target for Non-Small Cell Lung Cancer (NSCLC). In the cohort, EGFR mutations were limited to EGFR exon 19 deletions (ex19del) or exon 21 substitution (Leu858Arg) mutations. The FKBP10 positive rate in patients with EGFR mutations was 53% and 56% in patients without EGFR mutations (Fig. 1 D and E). The positive rate was similar (p = 0.817) between patients with EGFR mutations and those without (Fig. 1 D and E).

### Clinical factors affecting overall survival of lung adenocarcinoma patients with brain metastases

**Figure 2:**
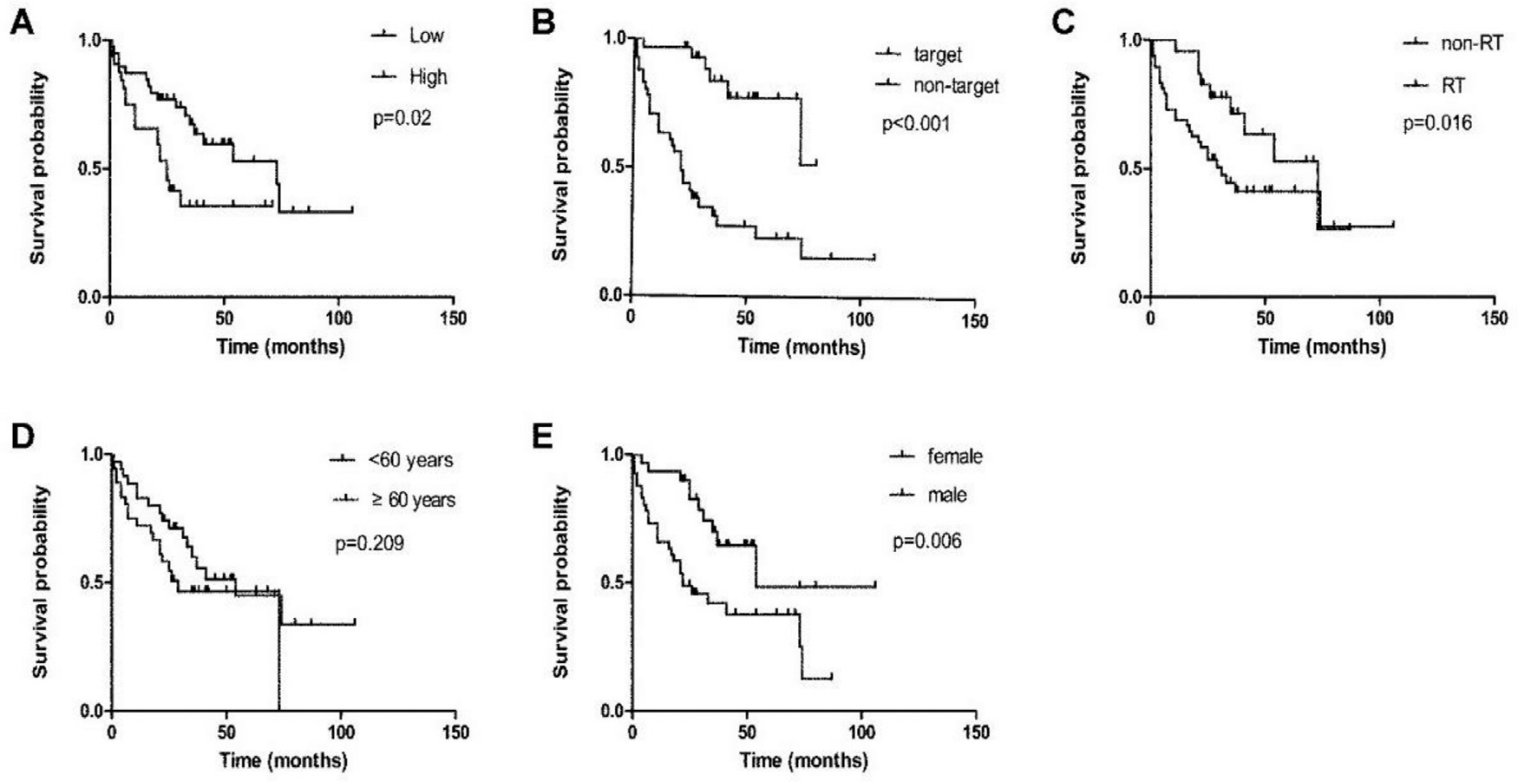
Factors affecting overall survival of lung adenocarcinoma patients with brain metastases. (A) FKBP10 expression. (B) Target therapy in lung adenocarcinoma brain metastases. (C) Radiotherapy (RT) in lung adenocarcinoma brain metastases. (D) Patient age in lung adenocarcinoma brain metastases. (E) Patient gender in lung adenocarcinoma brain metastases.

The median Overall Survival (OS) of the entire cohort was 41 months. In order to identify factors affecting OS in lung adenocarcinoma patients with brain metastases, the authors divided the enrolled patients into different subgroups according to age, sex, KPS, tumor size, location, target therapy and radiation treatment. According to the surgical results, patients received different postoperative treatments, including target therapy, radiation treatment, and observation. Thirty patients (42.3%) with EGFR mutations were treated with target therapy. These patients had a longer median OS than those who did not receive the targeted therapy (p < 0.001) (Fig. 2B). Patients who did not receive targeted therapy had a poor median OS of only 21 months. There were 23 patients (32.4%) who underwent postoperative radiotherapy in the cohort. They had a better median OS than those who did not receive radiotherapy (73 months vs. 31 months, p < 0.016) (Fig. 2C). The median OS of the older patient group (≥ 60 years) was 29 months, and the median OS of the younger group (< 60 years) was 54 months. However, univariate analysis showed that age was not associated with OS in patients with lung adenocarcinoma brain metastases (p=0.209) (Fig. 2D). There were 30 (42.3%) women and 41 (57.7%) men in the cohort. Univariate analysis showed that female patients had a longer median OS than male patients (54 months vs. 22 months, p = 0.006) (Fig. 2E). Besides, the authors did not find a statistical correlation between tumor size, location, and survival time in the present study. Because the enrolled patients had similar KPS (≥ 70) and the incidence of ALK fusion was low, the authors did not analyze the prognostic value of these two parameters.

### Prognostic value of FKBP10 in brain metastases

The authors analyzed FKBP10 expression using an immunohistochemical approach combined with a tissue microarray. Of the 71 patients with lung adenocarcinoma brain metastases, 39 (54.9%) had low and 32 (45.1%) had high FKBP10 expression. Kaplan-Meier analysis of 71 cases showed that the overall survival rates of patients with low FKBP10 expression were significantly higher than those with high positive FKBP10 staining (p = 0.02) (Fig. 2A). The median survival times of the patients with high FKBP10-expressing tumors and low FKBP10-expressing tumors were 25 months (n = 32) and 73 months (n = 39), respectively.

To identify independent prognostic factors affecting survival, the authors performed a multivariate Cox regression analysis. The following parameters are significant for survival in univariate analysis were sex, radiotherapy, targeted therapy, and FKBP10 expression. The results of the analysis are presented in Table 2. FKBP10 expression (p = 0.02, HR = 2.472, 95% CI [1.156, 5.289]), targeted therapy (p < 0.01, HR = 0.186, 95% CI [0.073, 0.477]), and radiotherapy (p = 0.006, HR = 0.330, 95% CI [0.149, 0.731]) were found to have an independent prognostic impact on survival of lung adenocarcinoma patients with brain metastases.

**Table 2:** Multivariate analysis of variables related to survival in lung adenocarcinoma patients with brain metastases.

Variables	Hazard ratio	95% CI	p-value
FKBP10	2.472	(1.156, 5.289)	0.02^a^
Target therapy	0.186	(0.073, 0.477)	< 0.01^a^
Radiotherapy	0.330	(0.149, 0.731)	0.006^a^
Gender	1.833	(0.827, 4.062)	0.136

a p < 0.05 was considered statistically significant.

### FKBP10 is also selectively expressed in primary lung adenocarcinoma and affects the overall survival and disease-free survival of patients

**Figure 3:**
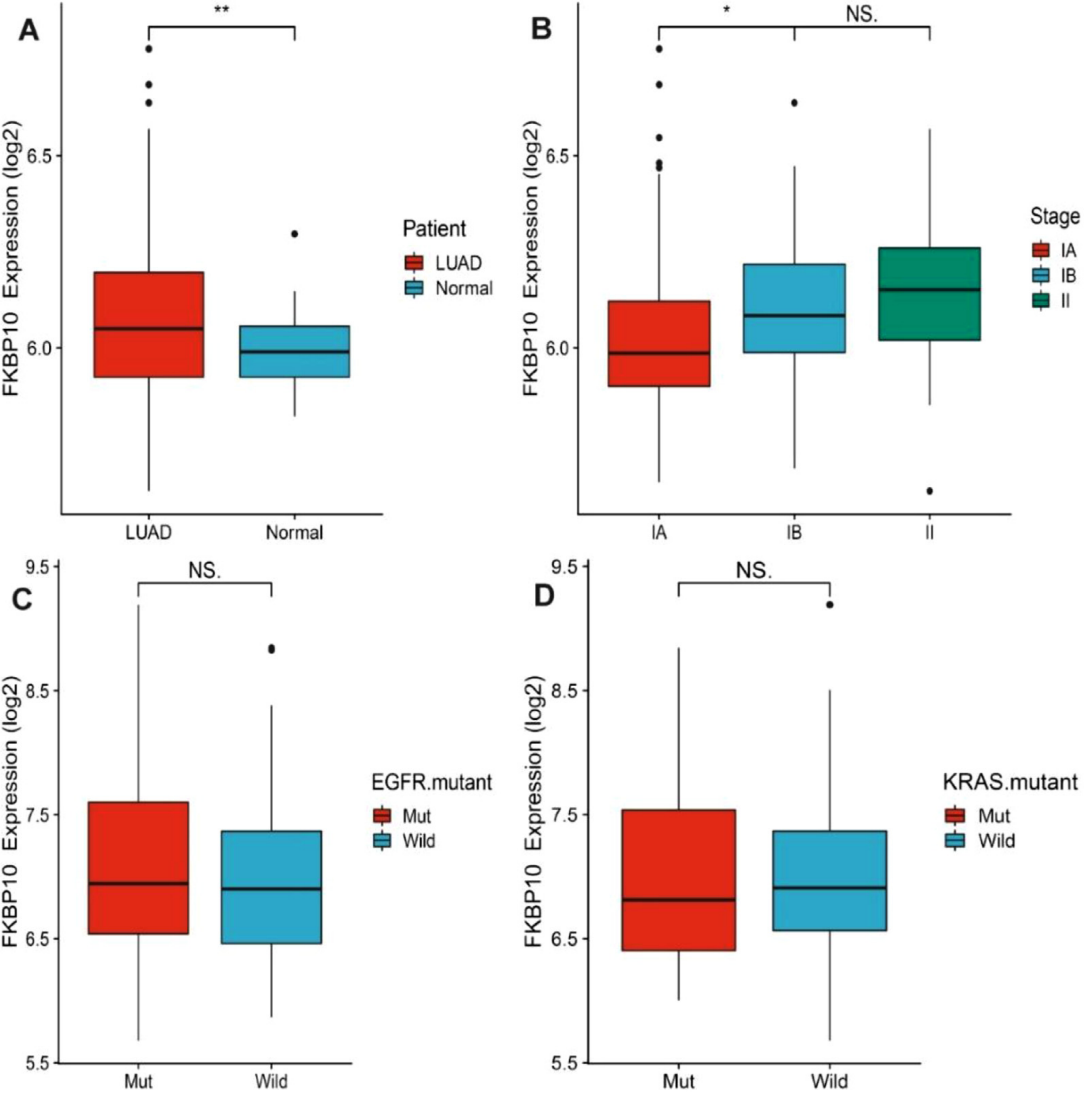
FKBP10 expression in primary lung adenocarcinoma. (A) The expression of FKBP10 between non-tumoral (Normal) and Lung Adenocarcinoma Specimens (LUAD) in the CANCERTOOL database and a Student *t*-test is performed in order to compare the mean gene expression between two groups. (B) The expression of FKBP10 among LUAD specimens of the indicated pathological stage in the CANCERTOOL database. Stage is indicated as IA, IB, II. An ANOVA test is performed in order to compare the mean among groups. (C) The expression of FKBP10 between LUAD specimens with wild type (non-mutant) and mutated Epithelial Growth Factor Receptor (EGFR mutant) in the CANCERTOOL database and a Student *t*-test is performed in order to compare the mean gene expression between two groups. (D) The expression of FKBP10 between LUAD specimens with wild type (non-mutant) and mutated KRAS (KRAS mutant) in the CANCERTOOL database and a Student *t*-test is performed in order to compare the mean gene expression between two groups. * Represents p < 0.05, ** Represents p < 0.01 and NS represents no statistically significant.

Considering the clinical significance of FKBP10 in brain metastases and the fact that no previous study has explored its expression in primary tumor lung adenocarcinoma, the authors have investigated the relevance of FKBP10 in lung adenocarcinoma. The authors first analyzed FKBP10 expression in human primary lung adenocarcinoma using the CANCERTOOL database. The authors found that higher FKBP10 expression was observed in lung adenocarcinoma than in normal lung tissue (p = 0.008) (Fig. 3A), which depended on tumor stage (p < 0.001) (Fig. 3B). Further analysis of the CANCERTOOL database revealed that the expression of FKBP10 was similar between lung adenocarcinoma bearing mutant KRAS, EGFR, and KRAS/EGFR non-mutants (Fig. 3 C and D), suggesting that the relevance of FKBP10 in lung cancer is not limited to a specific genotype.

**Figure 4:**
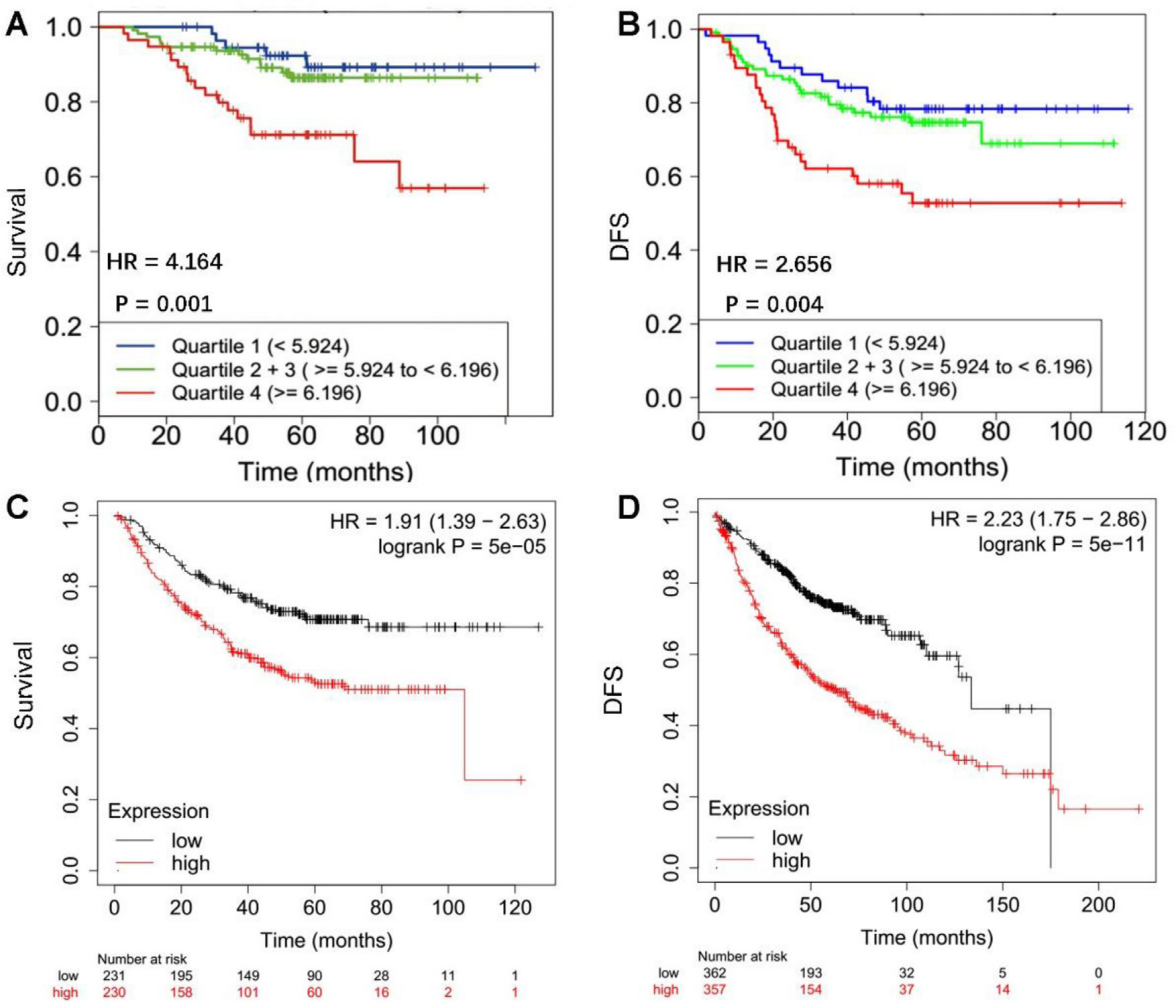
FKBP10 expression negatively correlates with Overall Survival (OS) and Diseases Free Survival (DFS) of patients with primary lung adenocarcinoma. (A and B) Data were obtained from CANCERTOOL database. Quartiles represent ranges of expression that divide the set of values into quarters. Quartile color code: Q1 (Blue), Q2 + Q3 (Green), Q4 (Red). Kaplan-Meier analysis and Mantel-Cox test were performed. (C and D) Data were obtained from KMPLOT. High expression: red, Low expression: black. Kaplan-Meier analysis and Mantel-Cox test were performed.

In addition, the authors analyzed the correlation between FKBP10 expression and patient survival. The authors found that FKBP10 expression was inversely correlated with the overall survival (p = 0.001) and disease-free survival (p = 0.004) time in patients with lung adenocarcinoma (Fig. 4 A and B) in the CANCERTOOL database. Finally, the authors validated the analysis results in the KMPLOT database and found the same conclusion that FKBP10 was inversely correlated with patients’ overall survival (p < 0.05) and disease-free survival (p < 0.05) (Fig. 4 C and D).

## Discussion

Although FKBP10, a peptidyl-prolyl-cis-trans-isomerase FK506-binding protein, has been reported as a potential cancer-related therapeutic target with a key biological role in tumor growth of NSCLC cells,^14^ the specific data on FKBP10 protein expression and its clinicopathological significance in NSCLC brain metastases remains unclear. Therefore, in the present study, the authors performed a survival analysis for lung adenocarcinoma patients with brain metastases who underwent surgical resection in the department and investigated the expression level and clinicopathological significance of FKBP10 in lung adenocarcinoma brain metastases.

First, the authors reviewed the pertinent preoperative information and follow-up information of patients in the cohort and found that the median overall survival was 41 months. According to a recent multi-institutional study, the overall survival of patients with lung adenocarcinoma brain metastases in the graded prognostic assessment 3.5–4.0 group is 47 months.^19^ Compared with prior reports,^20^^,^^21^ the survival of lung adenocarcinoma patients with brain metastases has improved significantly. The present results proved there has been progress in extending the survival of patients with brain metastases. The cause of survival improvement is multifactorial, in which surgery, radiotherapy, and targeted therapy play important roles. It is reported that surgical resection for brain metastases can rapidly reduce the symptomatic mass effect and vasogenic edema and offer prolonged survival when followed by adjuvant radiotherapy.^6^^,^^22^ In the cohort, radiotherapy was an independent prognostic factor for patient survival, and patients in the postoperative radiotherapy group had better median survival than the non-radiotherapy group (73 months vs. 31 months, p = 0.0161). This result indicates that the combination of surgery and adjuvant radiotherapy can improve survival in patients with brain metastases. The introduction of tyrosine kinase inhibitors has greatly improved the survival of lung cancer patients with EGFR mutations.^23^ In the present study, targeted therapy was positively correlated with a good prognosis (p < 0.0001). This is consistent with multi-institutional retrospective data showing the efficacy of tyrosine kinase inhibitors in patients with EGFR-mutated lung adenocarcinoma with brain metastases. The survival information of patients with lung adenocarcinoma brain metastases treated with a combination of neurosurgical resection and targeted therapy is limited. Previous studies have focused on targeted therapy as a major treatment for patients with brain metastases.^6^ Patients in the cohort had better survival results than those in a previous report.^19^ In our opinion, surgical resection could effectively reduce the tumor load and offer accurate tumor mutation information for targeted therapy, which may contribute to the prolonged survival of patients with brain metastases. Some studies reported that clinically actionable alterations present in brain metastases are frequently not detected in primary biopsies,^24^ which suggests that sequencing of primary biopsies alone may miss a substantial number of opportunities for targeted therapy.

FKBP10 was reported to be overexpressed in many cancers and not found in normal tissues around malignant tumors.^11^^,^^13^^,^^14^^,^^25^ In the present study, the authors found that FKBP10-positive cells were only detected in brain metastatic lesions of lung adenocarcinoma and not in non-tumor brain tissue. Moreover, the authors also investigated FKBP10 expression levels in primary lung adenocarcinoma using public databases. The results showed that primary lung adenocarcinoma tissue had higher FKBP10 expression than normal lung tissue (p = 0.008). Some researchers performed FKBP10 immunohistochemistry analysis in 32 cases of healthy lung tissue and 160 cases of NSCLC (80 cases of each squamous cell carcinoma and adenocarcinoma) and they found that FKBP10-positive cells were detected only in cancer lesions and not in the healthy parenchyma.^14^ The authors further analyzed the relationship between FKBP10 expression and EGFR mutation status and found that the expression of FKBP10 is similar between lung adenocarcinoma brain metastases bearing EGFR mutations and EGFR non-mutants. These lines of evidence indicate that FKBP10 may be a lung cancer-specific biomarker in primary lesions and brain metastases.

It been reported that FKBP10 plays an important role in tumorigenesis and development.^12^^,^^26^ However, the clinical implications of FKBP10 in lung adenocarcinoma brain metastases remain unclear. In this study, the authors found that FKBP10 expression has an independent prognostic impact on the survival of lung adenocarcinoma patients with brain metastases. For lung adenocarcinoma patients with brain metastases, high expression of FKBP10 predicts a worse survival time than for those with low or no expression. Survival analysis in public databases also indicates that FKBP10 is inversely correlated with the overall survival and disease-free survival of patients with primary lung adenocarcinoma. It was also reported that FKBP10 downregulation suppressed lung tumorigenesis.^14^ Thus, low FKBP10 expression may indicate less malignancy in lung adenocarcinoma brain metastases. It has been reported that nintedanib in combination with docetaxel is an effective second-line option for patients with advanced NSCLC previously treated with one line of platinum-based therapy, especially for patients with adenocarcinoma.^27^ Interestingly, nintedanib downregulates FKBP10 expression.^15^ Further studies are needed to investigate whether nintedanib could improve the prognosis of high FKBP10-expressing lung adenocarcinoma brain metastases.

### Limitation

Because the present study was retrospective and the time span was relatively large, there may be significant differences in the treatment regimens of patients. Additionally, the number of patients enrolled in the present study was relatively small, which may limit the statistical power and precision of the present results. These limitations may introduce bias into the present study and affect the accuracy and reliability of the results.

## Conclusion

In conclusion, the information the authors reported provides valuable insights for neurosurgeons managing patients with lung adenocarcinoma brain metastases. To the best of our knowledge, this is the largest cohort of surgical resections for lung adenocarcinoma brain metastases in the last 10 years. Our findings suggest that a combination of surgical resection, adjuvant radiotherapy, and precisely targeted therapy may benefit the survival of selected patients with lung adenocarcinoma brain metastases. In addition, the present study also demonstrates that FKBP10 is a novel biomarker for lung adenocarcinoma brain metastases, which is closely associated with survival time and may serve as a potential therapeutic target.

## Authors’ contributions

H-Q.C, J-H.W and S-H.W designed the study, analyzed the data, and revised the manuscript. Z-D.L and S-Q.W performed the experiments, analyzed the data and drafted the manuscript. S.L and J.H contributed materials, and collected clinical information.

## Ethics approval

This research was approved by the Ethics Committee of the Cancer Hospital, Chinese Academy of Medical Sciences (NCC2021C-516).

## Fundings

This study was supported by the CAMS Innovation Fund for Medical Sciences (n° 2021-1-I2M-012) and the National Natural Science Foundation of China (n° 82072803, 82103231).

## Conflicts of interest

The authors declare no conflicts of interest.
